# Maternal BHPF exposure as a risk factor for congenital heart disease in fetuses

**DOI:** 10.1093/nsr/nwaf553

**Published:** 2025-12-04

**Authors:** Guankai Zhan, Kunhui Su, Ruoan Jiang, Xiaoxiu Huang, Jiafeng Chen, Kao-Jung Chang, Mingjuan Jin, Baohua Li, Chih-Hung Hsu

**Affiliations:** Department of Obstetrics and Gynecology, Center for Genetic Medicine, the Fourth Affiliated Hospital of School of Medicine, and International School of Medicine, International Institutes of Medicine, Zhejiang University; Department of Environmental Medicine, Zhejiang University School of Medicine; Department of Obstetrics and Gynecology, Center for Genetic Medicine, the Fourth Affiliated Hospital of School of Medicine, and International School of Medicine, International Institutes of Medicine, Zhejiang University; Department of Environmental Medicine, Zhejiang University School of Medicine; Department of Obstetrics, Women’s Hospital, Zhejiang University School of Medicine; Department of Obstetrics, Women’s Hospital, Zhejiang University School of Medicine; Department of Obstetrics and Gynecology, Center for Genetic Medicine, the Fourth Affiliated Hospital of School of Medicine, and International School of Medicine, International Institutes of Medicine, Zhejiang University; Department of Environmental Medicine, Zhejiang University School of Medicine; Department of Medical Research, Taipei Veterans General Hospital; Department of Public Health, Second Affiliated Hospital, Zhejiang University School of Medicine; Department of Obstetrics, Women’s Hospital, Zhejiang University School of Medicine; Department of Obstetrics and Gynecology, Center for Genetic Medicine, the Fourth Affiliated Hospital of School of Medicine, and International School of Medicine, International Institutes of Medicine, Zhejiang University; Department of Environmental Medicine, Zhejiang University School of Medicine; Central Laboratory, Women and Children’s Hospital of Ningbo University

Congenital heart defects (CHDs) are among the most common congenital malformations in humans, yet only about 15% of cases can be traced to a clear genetic defect, posing challenges for effective clinical management [1–3]. Recent studies indicate that the internal human exposure levels of fluorene-9-bisphenol (BHPF) can induce significant cardiac developmental defects in animal models [4], and BHPF has been detected in various environmental media and human tissues [5,6]. Recognizing the implications of these findings, we undertook a clinical case–control study to investigate whether BHPF is associated with CHDs. From June 2024 to January 2025, 21 943 pregnant individuals underwent prenatal examinations at the Women’s Hospital, School of Medicine, Zhejiang University in Hangzhou, China. Among the fetuses of these pregnant women, 191 cases were diagnosed with CHDs, which included 20 cases of cardiac structural abnormalities, 1 case of cardiac rhythm abnormalities, and 170 cases of CHDs were excluded (including 46 cases who did not deliver in our hospital, 23 twin pregnancies with fetal cardiac anomalies, 8 with autoimmune diseases, 25 with chromosomal anomalies, 46 without chromosomal testing and 22 who did not wish to be enrolled). Finally, 21 valid CHD cases were catalogued as the CHD group, including 8 cases of ventricular septal defect, 2 cases of tetralogy of Fallot, 2 cases of Ebstein’s anomaly, 2 cases of persistent truncus arteriosus, 2 cases of

complete transposition of the great arteries, 1 case of hypoplastic left heart syndrome, 1 case of double outlet right ventricle, 1 case of complete atrioventricular septal defect, 1 case of pulmonary valve stenosis and 1 case of paroxysmal supraventricular tachycardia. As detailed in the Methods section, among the 21 enrolled cases, 57.2% were classified as severe CHDs and 43% resulted in pregnancy termination. These proportions closely mirror those observed in the overall CHD patient population at our center. Additionally, 42 control pregnant participants were recruited as the control group through 1:2 individual matching based on maternal age and pre-delivery body mass index (BMI) (Fig. 1a). The basic characteristics of these two groups are shown in Fig. 1b. There were no significant differences between the CHD and control groups in terms of demographic characteristics, lifestyle, pregnancy complications [hypertensive disorders of pregnancy (HDP), gestational diabetes mellitus (GDM), gestational anemia], and gestational external intervention factors (folic acid supplementation, assisted reproductive technology intervention and family history of coronary artery disease) (Fig. 1b). Maternal blood, amniotic fluid and umbilical cord blood were collected from the two groups using glass sampling tubes and the concentrations of 13 bisphenols including BHPF in the samples were determined by high performance liquid chromatography tandem mass spectrometry (LC-MS/MS)

**Figure 1.**
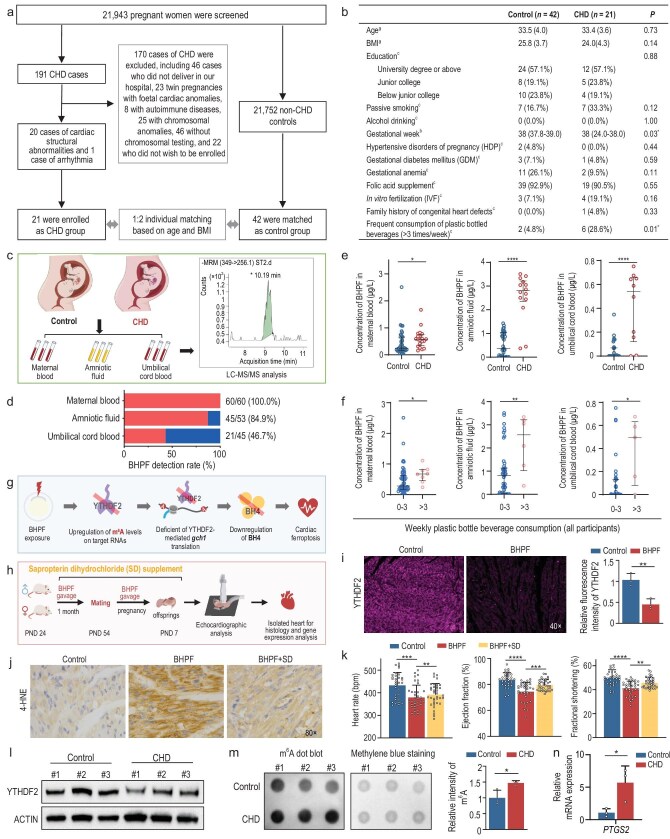
Association between maternal BHPF levels and fetal CHD and SD-mediated intervention for BHPF-induced CHDs in preclinical examination. (a) Enrolment of 42 controls and 21 CHD cases from 21 943 pregnant women for the case–control study. (b) Demographic and basic characteristics of the study participants. ^a^The results were presented as the mean (SD). ^b^The results were presented as median (interquartile range). ^c^The results were presented as *n* (%). *P < 0.05. (c and d) Schematic illustration of the collection and detection of maternal blood, amniotic fluid and umbilical cord blood in the CHD group and control group. The peak plot represents an example of BHPF detection by LC/MS-MS (c). The bar chart displays the detection rate of BHPF (d). (e) The concentration of BHPF in maternal blood, amniotic fluid and umbilical cord blood of the CHD group and the control group. *P < 0.05, ****P < 0.0001. (f) The concentration of BHPF in maternal blood, amniotic fluid and umbilical cord blood of participants who consume plastic-bottled beverages 0–3 times per week or more than 3 times per week. *P < 0.05, **P < 0.01. (g) Illustration: BHPF-mediated downregulation of YTHDF2 disrupts m^6^A homeostasis and promotes ferroptosis-induced cardiac defects. (h) Schematic diagram of the cross-generational BHPF exposure mice with SD intervention. (i) The changes of YTHDF2 level in offspring mice hearts upon BHPF exposure were detected by immunofluorescence with quantification. The magnification of microscope observation was 40×. **P < 0.01. (j) The rescue effect of the reduction of lipid peroxide 4-HNE level by SD in offspring mice hearts upon BHPF exposure. The magnification of microscope observation was 80×. (k) The rescue effect of heart rate, EF and FS by SD in offspring mice upon BHPF exposure. **P < 0.01, ***P < 0.001, ****P < 0.0001. (l) Western blot analysis of YTHDF2 protein levels in placentas of the CHD group and the control group. (m) The changes of m^6^A levels in placentas of the CHD group and the control group were detected by dot-blot with quantification. *P < 0.05. (n) The changes of *PTGS2* mRNA expression in placentas of the CHD group and the control group were detected by reverse transcriptase quantitative polymerase chain reaction (RT-qPCR). *P < 0.05.

(Fig. 1c). Despite the discovery of various bisphenol A (BPA) substitutes showing different toxic effects [4,7–9], 10 distinct BPA substitutes were detected in pregnant participants, with BHPF being 100% detected in maternal blood, 85% in amniotic fluid, and 47% in umbilical cord blood, respectively (Fig. 1d, Fig. S1 and Table S1). Although BHPF had high detection rates in both the CHD and control groups (Table S1), the median BHPF concentrations in maternal blood, amniotic fluid and umbilical cord blood of the CHD group were 0.58, 2.80 and 0.54 μg/L, respectively, which were significantly higher than those in the control group [0.25 (*P <* 0.05), 0.37 (*P <* 0.0001) and 0 μg/L (*P <* 0.0001), respectively] (Fig. 1e). In addition, among all pregnant participants, the concentrations of BHPF exhibited a significant positive correlation across the three sample types (*P <* 0.01) (Table S2). The above results reveal the prevalence of BHPF exposure and its accumulation in the pregnant population as a potential risk factor for CHDs.

Currently, BHPF is widely used in the manufacturing of plastic products. A questionnaire interview with all pregnant participants revealed that in the group who frequently consumed plastic-bottled beverages during pregnancy (>3 times per week), the median BHPF concentrations in maternal blood (0.68 μg/L), amniotic fluid (2.09 μg/L) and umbilical cord blood (0.16 μg/L) were significantly higher than those in the low-frequency group (0–3 times per week), which were 0.26 μg/L in maternal blood (*P <* 0.05), 0.74 μg/L in amniotic fluid (*P <* 0.01) and 0 μg/L in umbilical cord blood (*P <* 0.05) (Fig. 1f) in the CHD group. Categorical variables in the questionnaire survey were analyzed using Fisher’s exact test, which revealed a significant statistical difference in the percentage of pregnant individuals who frequently consume plastic-bottled beverages (>3 times per week) between the CHD (28.6%) and control (4.8%) groups. (*P <* 0.05) (Fig. 1b).

Recently, we identified that BHPF mediates early cardiac development defects by disrupting YTHDF2/m^6^A homeostasis, leading to the downregulation of GCH1 and subsequently reducing the production of tetrahydrobiopterin (BH4); as BH4 is a crucial ferroptosis inhibitor, this molecular cascade ultimately triggers cardiac ferroptosis and developmental abnormalities in animal models [4] (Fig. 1g). In this study, we demonstrated that BH4 supplementation (Fig. S2a) effectively repressed BHPF-induced ferroptosis (*ptgs2a* as an indicator of ferroptosis) (Fig. S2b) and cardiac defects (Fig. S2c–d) in zebrafish.

Notably, sapropterin dihydrochloride (SD), the synthetic form of BH4, has been clinically approved for treating patients with BH4 deficiency, such as hyperphenylalaninemia (HPA). Therefore, we explored whether SD could rescue BHPF-induced cardiac defects in offspring from BHPF-exposed parental mice (Fig. 1h). Consistent with previous studies [4], BHPF exposure led to the downregulation of YTHDF2 and GCH1 in the hearts of offspring from BHPF-exposed parental mice (Fig. 1i and Fig. S3a–b). Remarkably, SD reduced BHPF-induced accumulation of 4-hydroxy-2-nonenal (4-HNE), a lipid peroxidation metabolite and an indicator of ferroptosis (Fig. 1j and Fig. S3c), indicating that SD intervention can mitigate BHPF-induced cardiac ferroptosis. The results from echocardiography showed that the heart rate, ejection fraction (EF) and fractional shortening (FS) of the offspring mice were significantly decreased upon BHPF exposure (Fig. 1k), and SD intervention rescued these indicators of cardiac function (Fig. 1k). Histopathological observations revealed that BHPF induced myocardial inflammatory infiltration (Fig. S3d) and collagen fibrosis (Fig. S3e) in the offspring mice, whereas the SD intervention markedly suppressed these histopathological changes (Fig. S3d–e). Notably, unlike the observations in offspring, the hearts of the parental mice were not significantly affected by BHPF exposure or SD intervention (Fig. S3f).

To investigate the conservation of the BHPF-induced YTHDF2/m^6^A–ferroptosis axis, we examined the levels of key molecules in the placental tissue (infant side) across two groups: three representative cases from the CHD group with higher BHPF concentrations (2.50, 3.04 and 2.86 μg/L) and three representative cases from the control group with lower BHPF concentrations (0, 0.01 and 0.02 μg/L). We observed a downregulation of YTHDF2 (Fig. 1l), an upregulation of m^6^A RNA modifications (Fig. 1m), and elevated *PTGS2* mRNA (an indicator of ferroptosis) levels (Fig. 1n) in the placental tissue (infant side) from the CHD group. These findings suggest that the molecular mechanism underlying BHPF-induced defects is conserved from zebrafish to mice to humans.

Due to BPA’s multiple toxicities, major economies (such as the USA, the EU, China and Canada) have restricted its use in infant products since 2010–2012, with the EU even enacting Commission Regulation (EU) 2024/3190 to ban it (excluding exemptions). Consequently, BPA substitutes are widely used. Including BHPF, 10 distinct BPA substitutes are detected in pregnant participants (Fig. 1a–d and Fig. S1), which reveal that post-BPA restrictions, numerous BPA substitutes, without systematic safety evaluations, have proliferated unchecked.

Pregnant individuals consuming plastic-bottled beverages frequently showed higher BHPF levels in maternal serum, amniotic fluid and cord blood (Fig. 1f), with greater prevalence in CHD cases (Fig. 1b). This suggests that (i) BHPF could cross the placental barrier, leading to the accumulation of BHPF in offspring-related tissues (detected in amniotic fluid and cord blood) and intergenerational exposure; and (ii) plastic bottle use may be a significant exposure route and CHD risk factor.

Notably, BHPF concentrations were markedly elevated in amniotic fluid (median: 2.80 μg/L in CHD cases vs. 0.37 μg/L in controls), exceeding levels in umbilical cord blood (0.54 vs. 0 μg/L) and maternal serum (0.58 vs. 0.25 μg/L) (Fig. 1e). While the metabolic kinetics of BHPF remain unclear, previous studies suggest that the structurally similar BPA is primarily excreted via the kidneys and urine [10]. Additionally, BPA can undergo glucuronidation in the maternal proximal intestine and liver, then crossing the placental barrier and accumulating in amniotic fluid [11,12]. Therefore, both maternal sources and fetal urinary excretion may contribute to the progressive accumulation of BHPF in amniotic fluid, leading to higher BHPF concentrations in the environment surrounding the fetus compared to other parts of the maternal body. In parallel, the fetus regularly swallows amniotic fluid, further increasing its direct BHPF exposure and suggesting a greater risk to fetal development than to the pregnant individual. This may correspond to the observation that, while BHPF exposure resulted in significant cardiac defects in the offspring of exposed mice, no apparent cardiac defects were observed in the parental mice (Fig. 1k and Fig. S3f). Together, these findings suggest that the high concentration of BHPF in amniotic fluid could be the critical factor in causing fetal CHDs.

According to the American College of Medical Genetics and Genomics guidelines, SD is classified as a class C medication for use in pregnancy when the benefits outweigh the risks [13]. While SD shows a potential intervention, its therapeutic application requires comprehensive preclinical evaluation, including optimization of dosage regimens, administration frequency and delivery methods.

By elucidating the connection between maternal BHPF accumulation and CHD pathogenesis, this study provides valuable insight into the role of environmental pollutants in congenital heart disease development. In addition to its m^6^A-dependent cardiac defective effects, BHPF has been shown to exert anti-estrogenic activity [6], and lower estrogen is linked to various cardiac diseases [14]. The potential role of BHPF-induced estrogen suppression in CHD development merits further investigation. Together, this study underscores the necessity of extensive, multi-regional epidemiological studies to assess the broader implications of BHPF exposure. At the current stage, the use of BHPF should be strictly regulated, especially in maternal and infant products, to break the ‘vicious cycle of BPA substitutes’ before it triggers a preventable health crisis.

## Supplementary Material

nwaf553_Supplemental_Files
