# Supplementary material for: Maternal BHPF exposure as a risk factor for congenital heart disease in fetuses
Source: Natl Sci Rev. 2025 Dec 4;12(12):nwaf553. doi: 10.1093/nsr/nwaf553 (PMC12743451; doi:10.1093/nsr/nwaf553)
Supplement: nwaf553_Supplemental_Files [file nwaf553_supplemental_files.zip › NSR_MS-2025-1039.R1_Supplemental Information.docx]

Maternal BHPF Exposure as a Risk Factor for Congenital Heart Disease in Fetuses

Supplementary material

**Methods**

**Study Design and Participants**

From June 2024 to January 2025, we screened 21,943 pregnant individuals undergoing prenatal examinations at the Women’s Hospital (from Hubin Campus in Shangcheng District and [Qianjiang Campus](https://zju.womanhospital.cn/index/article/maps/pid/maps.html) in Xiaoshan District), School of Medicine, Zhejiang University in Hangzhou, China, identifying 191 cases of fetal congenital heart disease (CHD) through experienced ultrasound physicians. After excluding 46 cases that did not deliver at our hospital, 23 twin pregnancies with fetal cardiac anomalies, 8 cases with autoimmune diseases, 25 cases with chromosomal anomalies, 46 cases without chromosomal testing, and 22 cases that declined participation, we recruited 21 cases with isolated congenital heart defects as the CHD group. According to disease severity, fetal CHD cases in mainland China are typically classified into four types. Type I is generally considered a physiological variation and was therefore not included in our study. At our center, the distribution of CHD types II, III, and IV was approximately 40%, 47%, and 13%, respectively. The pregnancy termination rates for severe congenital CHD were 73% for type III and 92% for type IV cases. Consequently, the rate of prenatally diagnosed severe CHD was approximately 60%, comprising 47% type III and 13% type IV cases. Among the 21 cases enrolled in our study, 9 were classified as type II CHD (42.8%), 11 as type III (52.4%), and 1 as type IV (4.8%). Of these, 8 of the 11 type III cases and the single type IV case resulted in pregnancy termination.

Additionally, 42 healthy controls were included through 1:2 individual matching based on maternal age and pre-delivery BMI. The control fetuses were free from congenital heart disease, other structural malformations, and any growth disorders. While some control mothers had gestational diabetes (7.1%) or hypertensive disorders of pregnancy (4.8%), all were managed with non-pharmacological interventions (e.g., exercise). The participants were kindly signed an informed consent, completed a questionnaire, and denoted samples of maternal blood, umbilical cord blood amniotic fluid and placenta during delivery. A one-on-one questionnaire survey was conducted, including basic demographic data, environmental factors, disease history, and education.

**Statistical Analysis**

Continuous variable was analyzed by Student's t test when the data follows a normal distribution. or Mann−Whitney U-rank sum test when the data follows a normal distribution. Spearman’s correlation analysis was used to examine the correlation of BHPF concentration among maternal blood, amniotic fluid and umbilical cord blood. Categorical variables collected by questionnaire survey was analyzed by Fisher's exact test. Statistical analysis was assessed by IBM SPSS Statistics 27. The two-side probability value less than 0.05 was considered as statistically significant.

**Concentration Measurement of Bisphenols**

The 200 μL samples were stored in glass tubes at -80°C until analysis. Four volumes of HPLC grade methanol were added, samples were mixed for 3 min then centrifuged at 4 000 rpm for 30 min. Supernatant solutions from each sample were transferred and removed for LC-MS/MS analysis. All analytical procedures were checked for precision reproducibility, blank contamination and linearity. We employed the external standard method to establish the calibration curve for BHPF quantification. Standard solutions of BHPF were prepared at concentrations of 0.05 ppb, 0.1 ppb, 0.5 ppb, and 1 ppb.

The calibration curve exhibited good linearity, with a linear equation of y = 22636.24x + 266.31, where y represents the instrument response value and x represents the BHPF concentration (ppb, μg/L), R^2^ = 0.9955. The Limit of Detection (LOD) was calculated as 0.01 pg/L and the Limit of Quantification (LOQ) was calculated as 0.2 pg/L.

About blank tests: maternal blood was collected using glass coagulation-promoting vacuum blood collection tubes. LC-MS/MS analysis showed a response value of 0, indicating the absence of BHPF. Amniotic fluid and umbilical cord blood were collected using polypropylene syringes. The detected LC-MS/MS response values for these samples were 1944.5±187.5. The concentrations of BHPF presented in the article for amniotic fluid and umbilical cord blood were obtained by subtracting the background value contributed by the syringes. The matrix effect was 98.42%±6.22% for maternal blood, 96.840%±1.827% for amniotic fluid, and 97.441%±0.267% for umbilical cord blood, indicating the absence of a significant matrix effect. Detailed parameters for analyzing the targeted compounds by LC-MS/MS are listed in the Table S3.

**Animal BHPF Exposure Procedure**

CD-1 mice were obtained from Shanghai Slac Laboratory Animal CoLTD. Mice were kept in a controlled environment (22±2 °C, 55±5% humidity, 12 light/dark cycle) under standard conditions with water and food ad libitum. BHPF was dissolved in 0.1% DMSO (0.1% DMSO were used as a control). CD-l mice were intragastrically administered with BHPF (70 μg kg^-1^ bw 2-d^-1^) from postnatal day (PND) 24 for 1 month then mating, and F_1_ offspring birth to PND 7 were subjected to echocardiographic detection (parental mice gavage dose of 70 μg kg^-1^ bw 2-d^-1^, with BHPF concentrations of 0.05-0.1 μg/L in the serum). Sex-matched littermates were used for experiments in order to test a single variable. Then, these offspring were euthanized, and the heart were isolated for histological as well as gene expression analysis. Adult transgenic zebrafish Tg(*lcr*:eGFP) used in this study were raised and maintained at 28.5°C and on a 14 h light/10 h dark cycle at zebrafish core facilities of Zhejiang University School of Medicine. Zebrafish embryos were subjected to indicated treatments at 4 hpf. 10 μM final concentration of BHPF was dissolved to a storage solution of 10 mM with DMSO (Sigma, D2650) as solvent, and 0.1% DMSO were performed with as control. The specific final concentration of Tetrahydrobiopterin (BH4) (MCE, HY-107383) co-treatment was 200 μM.

**Morphological Observation and Heart Rate Evaluation for Zebrafish Lines**

For morphological observation, drugs exposed zebrafish embryos were carefully visualized and imaged by SMZ18 (Nikon) microscope at 48 hpf. For cardiac evaluation, videos of heart activity were recorded from zebrafish placed in lateral recumbency and analyzed by DanioScope (Noldus).

**Western Blot and Antibody**

To get the whole cell lysates, samples were cracked at 4°C by RIPA lysis buffer (150 mM NaCl, 50 mM Tris-HCl, 5 mM EDTA, 5% Glycerol, 1% TritonX-100, 0.1% SDS) with protease inhibitors (Bimake, B14001). Ultrasonication was employed to ensure thorough lysis for extraction. Then the extractions were centrifugated for 10 min at 14,000 g at 4°C to obtain cell lysates. After denaturation, cell lysates were separated on a 10% polyacrylamide gel (GenScript, M00656), transferred onto a NC membrane (GVS, 1215458). Membranes were blocked by 5% skim milk powder for 1h at room temperature. After primary and secondary antibody incubation, target protein stripes were imaged on the Tanon-5200 Chemiluminescent Imaging System (Tanon Science & Technology). Actin was used as the internal control. The information of antibodies used in this study were shown in the Table S4.

**RNA extraction and quantitative RT-PCR**

The total RNA of cells was extracted with RNAiso Plus (Takara, 9109) in line with the protocols of manufacturer. The cDNA was synthesized by PrimeScriptTM RT reagent Kit with gDNA Eraser (Takara, RRO47A) according to the manufacturer’s instructions. Light Cycler 480 (Roche) was used to detect the expression level of target genes using 2×Universal SYBR Green Fast qPCR Mix (ABclonal, RK21203). The examined genes were normalized to β-Actin mRNA expression. The specific primer sequences of examined genes are listed in Table S5.

**Immunofluorescence Staining (IF)**

Mice hearts were fixed in 4% paraformaldehyde overnight at 4°C. After gradient dehydration with ethanol, the samples were dealt with Xylene and then embedded with paraffin. Paraffin sliced samples were deparaffinized through xylene and 95% ethanol. After retrieving the tissue sections antigen with sodium citrate solution, sections were washed by PBS 3 times, and blocking the samples with 5% BSA blocking solution for 1 h at 4°C, followed by incubating overnight at 4°C with primary antibody. After PBST wash, the samples were incubated with secondary antibody for 1 h at 37°C. Finally, nuclei were stained with DAPI (Beyotime, C1002). The fluorescent signals were visualized under bx63 (Olympus) microscope. The information of antibodies used in this study were shown in the Table S4.

**Hematoxylin & Eosin Staining and Sirius Red Staining**

Mice hearts were embedded with paraffin and sliced, the tissue sections were stained with hematoxylin & eosin (Beyotime, C0105M) or sirius red (Macklin, 771862) and then imaged by bx63 (Olympus, Japan) microscope. Sirius red staining was used to measure collagen deposits.

**Cardiac Function Evaluation**

For F_1_ mice offspring cardiac function evaluation, echocardiography was performed using the Vevol100® Imaging System and VevoImaging Station (VisualSonics) equipped with high frequency transducers. Heart rate per minute, ejection fraction and fractional shortening were analyzed as cardiac function evaluation indicators.

**Ethical Considerations**

The human samples were obtained after appropriate ethical approval from the Medical Ethics Committee of the Women's Hospital, School of Medicine, Zhejiang University (IRB-20240156-R), and all participants have obtained written informed consent. The animal experiments have been reviewed and approved by the Laboratory Animal Welfare and Ethics Review Committee of Zhejiang University (ZJU20230144).

**Figure S1. The detection rate of 12 bisphenols in maternal samples**

1. The detection rate of 12 bisphenols in maternal blood, amniotic fluid and umbilicalcord blood in the recruited pregnant participents (Fig. 1a). n denotes the number of pregnant participents.
2. The concentrations of 12 bisphenol analogues detected in maternal samples from both the control group and the CHD group. The numbers displayed below the groups represent the median of each group. N. D means not detected. ^*^*P*<0.05, ^**^*P*<0.01, ^***^*P*<0.001

**Figure S2. BH4 rescues BHPF-induced cardiac defects in zebrafish model**

(a) Schematic diagram of BHPF-induced cardiac defects in zebrafish model with BH4 supplement.

(b) The rescue effect of *ptgs2a* mRNA expression by BH4 in zebrafish upon BHPF exposure. ^****^*P<*0.0001

(c) The rescue effect of heart rate by BH4 in 48 hpf zebrafish embryos upon BHPF exposure. ^****^*P<*0.0001

(d) The rescue effect of pericardial congestion by BH4 in zebrafish upon BHPF exposure with quantification. The magnification of microscope observation was 50x.

**Figure S3. SD rescues BHPF-induced cardiac defects in mice model**

(a) The changes of YTHDF2 and GCH1 protein levels in offspring mice hearts upon BHPF exposure were detected by western-blot.

(b) The changes of GCH1 level in offspring mice hearts upon BHPF exposure were detected by immunofluorescence with quantification. The magnification of microscope observation was 40x. ^**^*P<*0.01

(c) The immunohistochemical images of 4-HNE was quantified as a histogram. ^****^*P<*0.0001

(d-e) Representative images of heart sections stained by hematoxylin and eosin (H&E) to observe inflammatory responses (d) and sirius red to observe myocardial fibrosis (e). Red arrows indicate inflammatory infiltration, and blue arrows indicate collagen fiber deposition. The magnification of microscope observation was 40x.

(f) The effects of BHPF exposure and SD supplementation on heart rate, ejection fraction, and fractional shortening in parental mice. ns denotes no significance

Supplemental Tables

**Table. S1 The detection rates of 13 bisphenols in maternal samples between the control group and the CHD group.**

|  | **Maternal blood** | | | **Amniotic fluid** | | | **Umbilical cord blood** | | |
| --- | --- | --- | --- | --- | --- | --- | --- | --- | --- |
|  | **Control** | **CHD** | ***P*** | **Control** | **CHD** | ***P*** | **Control** | **CHD** | ***P*** |
| BHPF | 100.0% | 100.0% | 1.000 | 79.5% | 100.0% | 0.069 | 34.3% | 90.0% | 0.002^**^ |
| BPA | 90.0% | 100.0% | 0.291 | 87.2% | 100.0% | 0.309 | 94.3% | 100.0% | 1.000 |
| BPAF | 42.5% | 70.0% | 0.058 | 30.8% | 85.7% | <0.001^***^ | 22.09% | 60.0% | 0.049^*^ |
| BPAP | 0% | 0% | / | 7.7% | 78.6% | <0.001^***^ | 0% | 0% | / |
| BPB | 0% | 0% | / | 5.1% | 7.1% | 1.000 | 0% | 0% | / |
| BPC | 0% | 0% | / | 0% | 0% | / | 0% | 0% | / |
| BPE | 25.0% | 0% | 0.023^*^ | 7.7% | 0% | 0.557 | 22.9% | 30.0% | 0.687 |
| BPF | 0% | 0% | / | 0% | 0% | / | 0% | 0% | / |
| BPM | 90.0% | 100.0% | 0.291 | 92.3% | 100.0% | 0.557 | 88.6% | 100.0% | 0.561 |
| BPP | 100.0% | 100.0% | / | 92.3% | 100.0% | 0.557 | 88.6% | 100.0% | 0.561 |
| BPS | 0% | 0% | / | 0% | 0% | / | 0% | 0% | / |
| BPSIP | 95.0% | 100.0% | 0.548 | 66.7% | 100.0% | 0.012^*^ | 77.1% | 90.0% | 0.659 |
| BPZ | 52.5% | 35.0% | 0.274 | 97.4% | 35.7% | <0.001^***^ | 97.1% | 90.0% | 0.399 |

^*^*P*<0.05, ^**^*P*<0.01, ^***^*P*<0.001

**Table S2. The correlations analysis of BHPF concentration in maternal blood, amniotic fluid and umbilical cord blood of CHD group and control group.**

|  | **Maternal blood** | **Amniotic fluid** | **Umbilical cord blood** |
| --- | --- | --- | --- |
| Maternal blood | 1.00 | 0.80^**^ | 0.55^**^ |
| Amniotic fluid | 0.80^**^ | 1.00 | 0.63^**^ |
| Umbilical cord blood | 0.55^**^ | 0.63^**^ | 1.00 |

The results were represented by correlation coefficient. ^**^ *P<*0.01

Table S3. Detailed parameters for LC/MS/MS.

| **Compound** | **Quantitative ion pair** | **Qualitative ion pair** |
| --- | --- | --- |
| BHPF | 349→256.1 | 349→215.1 |
| BPA | 227.3→212 | 227.3→212 |
| BPAF | 335→265 | 335→265 |
| BPAP | 289→274.1 | 289→274.1 |
| BPB | 241→212 | 241→212 |
| BPC | 279→35 | 279→35 |
| BPE | 213→198.1 | 213→198.1 |
| BPF | 199→93.1 | 199→93.1 |
| BPM | 345→251.1 | 345→330.1 |
| BPP | 345→330.1 | 345→207.8 |
| BPS | 249→108 | 249→108 |
| BPSIP | 291→248 | 291→184 |
| BPZ | 267→223 | 267→223 |

Table S4. Primary and secondary antibodies

Used for western blotting (WB), immunofluorescence (IF), immunohistochemistry (IHC) and m^6^A dot-blot.

| **Antibody** | **Source** | **Catalog#** | **Dilutions** |
| --- | --- | --- | --- |
| ACTIN | Proteintech | 20536-1-AP | WB 1:1000 |
| YTHDF2 | Proteintech | 24744-1-AP | WB 1:1000  IF 1:500 |
| GCH1 | Abclonal | A10616 | WB 1:1000  IF 1:500 |
| m^6^A | Abclonal | A17924 | Dot-blot 1:1000 |
| 4-HNE | Invitrogen | MA5-27570 | IHC 1:50 |
| Alexa Fluor 647 AffiniPure Goat Anti-Mouse IgG (H+L) | Jackson ImmunoResearch | 111-605-003 | IF 1:1000 |
| Anti-mouse HRP | Beyotime | A0216 | WB 1:5000 |
| Anti-rabbit HRP | Beyotime | A0208 | WB 1:3000 |

**Table S5. Primers used for cDNA amplification and RT-qPCR.**

| **Name** | **Forward (5'-3')** | **Reverse (5'-3')** |
| --- | --- | --- |
| Human-PTGS2 | TTGCATTCTTTGCCCAGCAC | ACCGTAGATGCTCAGGGACT |
| Human-β-Actin | CCCTGGAGAAGAGCTACGAG | CGTACAGGTCTTTGCGGATG |
| Mice-*ptgs2a* | CACACATGGCATTCGCAACA | TGCATTGAAAGATTGATAGCGCA |
| Mice-*β-actin* | GGCTGTATTCCCCTCCATCG | AGTTGGTAACAATGCCATGT |
