# Supplementary figures and images for: Maternal BHPF exposure as a risk factor for congenital heart disease in fetuses
Source: Natl Sci Rev. 2025 Dec 4;12(12):nwaf553. doi: 10.1093/nsr/nwaf553 (PMC12743451; doi:10.1093/nsr/nwaf553)

# Figure S1

**a**

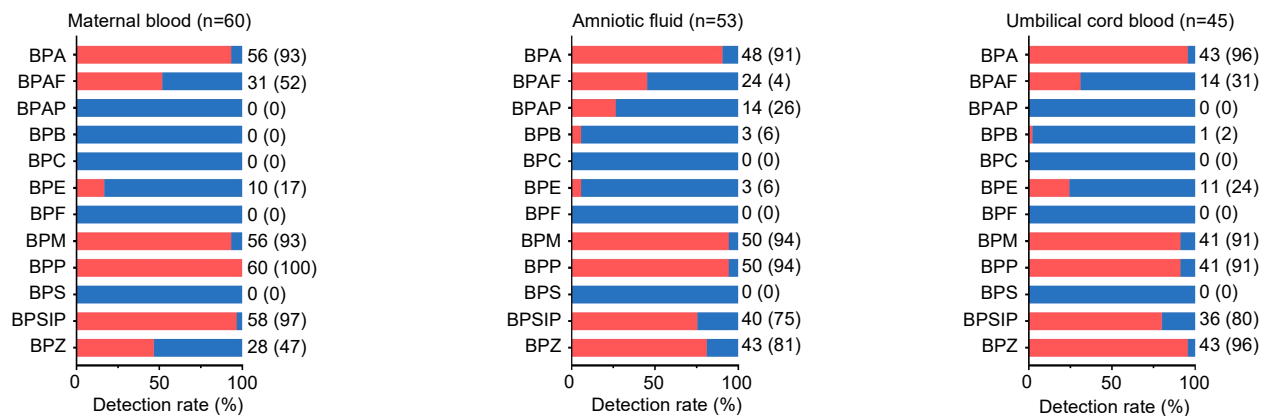

**b**

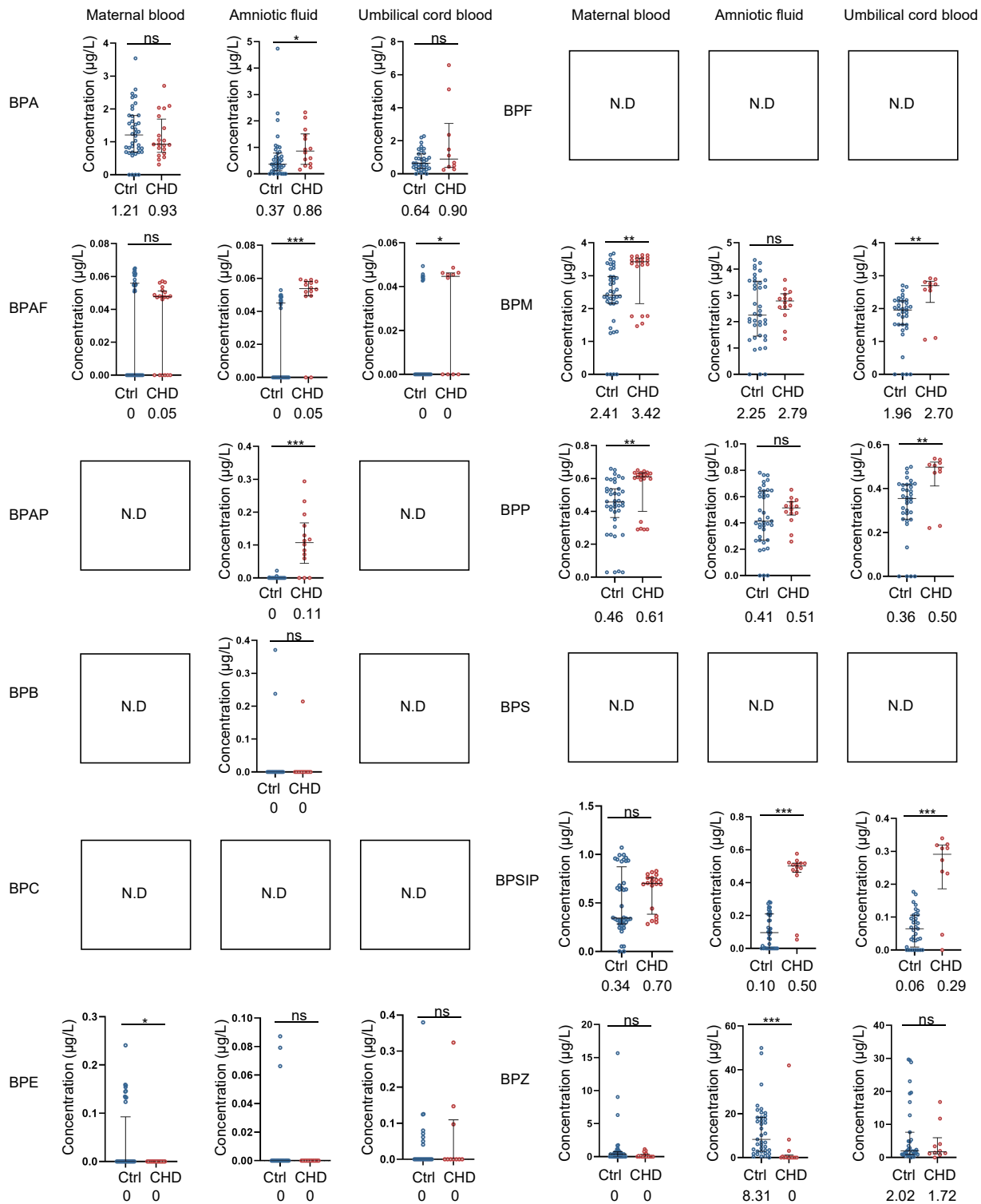

Figure S2

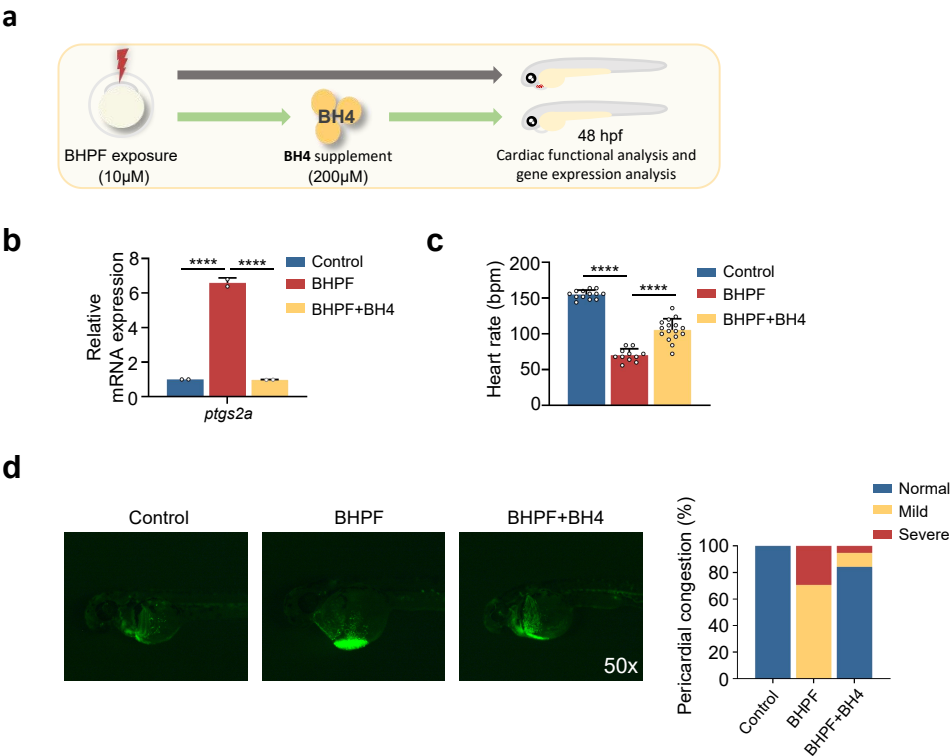

Figure S3

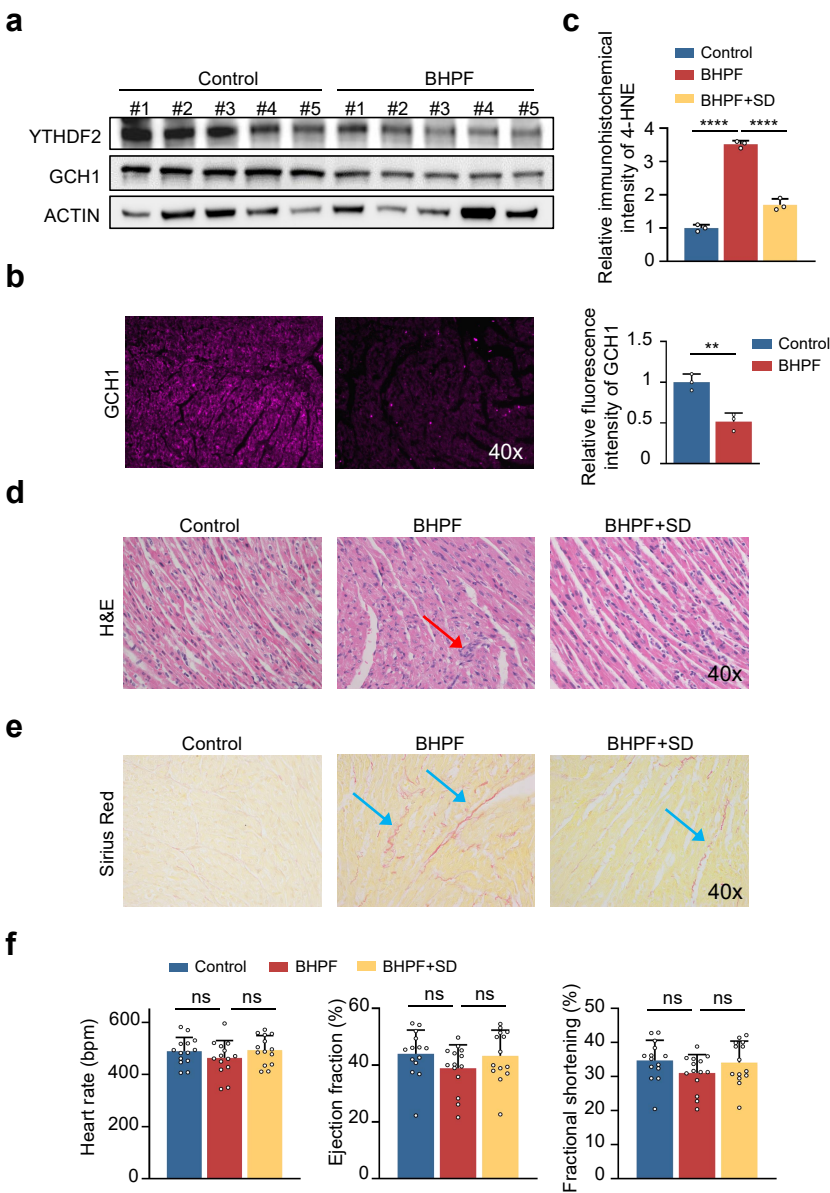

Supplement: nwaf553_Supplemental_Files [file nwaf553_supplemental_files.zip › Supplementary Figure.pdf]
